# Bee Venom Acupuncture Attenuates Oxaliplatin-Induced Neuropathic Pain by Modulating Action Potential Threshold in A-Fiber Dorsal Root Ganglia Neurons

**DOI:** 10.3390/toxins12120737

**Published:** 2020-11-24

**Authors:** Ji Hwan Lee, Juan Gang, Eunhee Yang, Woojin Kim, Young-Ho Jin

**Affiliations:** 1 Department of Physiology, College of Korean Medicine, Kyung Hee University, Seoul 02453, Korea; mibdna@khu.ac.kr; 2 Department of Science in Korean Medicine, Graduate School, Kyung Hee University, Seoul 02453, Korea; 3 Department of East-West Medicine, Graduate School, Kyung Hee University, Seoul 02453, Korea; khan2296@naver.com; 4 Department of Physiology, College of Medicine, Kyung Hee University, Seoul 02453, Korea; dmsgmls317@gmail.com

**Keywords:** action potential, allodynia, bee venom acupuncture, oxaliplatin, voltage-gated sodium channel

## Abstract

Oxaliplatin is a third-generation platinum-based chemotherapeutic drug widely used in colorectal cancer treatment. Although potent against this tumor, it can induce cold and mechanical allodynia even after a single injection. The currently used drugs to attenuate this allodynia can also cause unwanted effects, which limit their use. Bee venom acupuncture (BVA) is widely used in Korean medicine to treat pain. Although the effect of BVA on oxaliplatin-induced neuropathic pain has been addressed in many studies, its action on dorsal root ganglia (DRG) neurons has never been investigated. A single oxaliplatin injection (6 mg/kg, intraperitoneally) induced cold and mechanical allodynia, and BVA (0.1 and 1 mg/kg, subcutaneous, ST36) dose-dependently decreased allodynia in rats. On acutely dissociated lumbar 4–6 DRG neurons, 10 min application of oxaliplatin (100 μM) shifted the voltage-dependence of sodium conductance toward negative membrane potentials in A- but not C-fibers. The resting membrane potential remained unchanged, but the action potential threshold decreased significantly compared to that of the control (*p* < 0.05). However, 0.1 μg/mL of BVA administration increased the lowered action potential threshold. In conclusion, these results suggest that BVA may attenuate oxaliplatin-induced neuropathic pain by altering the action potential threshold in A-fiber DRG neurons.

## 1. Introduction

Oxaliplatin is a chemotherapeutic drug widely used in the treatment of advanced metastatic colorectal cancer [1], which is the second highest cause of death due to cancer after lung carcinoma [2]. Despite its effect on tumor size reduction, its use can be limited due to peripheral neuropathy rapidly occurring after infusion in up to 80–90% of treated patients. This peripheral neuropathy is characterized by a glove-and-stocking distribution sensory loss, paresthesia, and dysesthesia, which can be triggered and aggravated by exposure to cold [3]. To prevent or attenuate this oxaliplatin-induced neuropathic pain, various strategies and drugs have been proposed such as “stop and go” strategy, Ca/Mg infusion, glutathione, and duloxetine. Recently, the American Society of Clinical Organization guidelines [4] and the Journal of the American Medical Association [5] recommended duloxetine, which is a well-established anticonvulsant and analgesic drug, to mitigate oxaliplatin-induced pain. However, even duloxetine has been reported to cause side effects, such as dizziness and headache, demonstrating the need for the development of alternative strategies [6].

For the past several years, our group has focused on understanding the underlying mechanism of oxaliplatin-induced neuropathic pain and on finding an effective treatment method. The effects of well-known analgesic drugs such as morphine [7], gabapentin [8], and duloxetine [9] have been assessed along with herbal medicines [10,11,12], acupuncture [8,13], and bee venom acupuncture (BVA) [7,14,15,16]. Bee venom (BV) is composed of peptides, enzymes, amines, and non-peptide components that have a variety of pharmaceutical properties [17]. Subcutaneous injection of BV at acupoints, BVA, is a widely used method in Korean medicine for the treatment of different kinds of pain such as musculoskeletal [18,19] and arthritis pain [20]. In our previous studies, BVA significantly attenuated cold and mechanical allodynia induced by oxaliplatin. Moreover, BVA induced an additive effect when simultaneously injected with morphine by increasing the action time in mice injected with a chemotherapeutic agent [8]. Furthermore, three weeks of treatment with BVA in patients with chemotherapy-induced neuropathic pain also resulted in positive effects [21], implying the efficacy of BVA against oxaliplatin-induced neuropathic pain.

The underlying mechanism of oxaliplatin-induced neuropathic pain remains unclear; however, oxaliplatin is known to directly affect the dorsal root ganglia (DRG) neurons, whereas in the central nervous system, its direct effect is smaller because of the limited permeability of oxaliplatin through the blood–brain barrier (BBB) [22]. Upon excitation, DRG neurons can transmit sensory signals such as pain to the spinal cord by generating action potentials [23]. The excitability of a neuronal cell depends upon various factors, but ion channels play a critical role. Oxaliplatin is reported to induce an immediate effect on the axonal excitability rather than cause structural changes, suggesting that ion channels in DRG neurons may be involved [24]. Indeed, abnormalities in the functions of voltage-gated potassium ion and calcium ion (K_v_ and Ca_v_, respectively) channels have been reported [25]; however, sodium ion (Na_v_) channels may play a major role in oxaliplatin-induced neuropathic pain, as these channels determine the occurrence and development of neuropathic pain in primary sensory neurons [26,27,28]. In addition, sodium channel blockers such as carbamazepine significantly attenuated oxaliplatin-induced neuropathic pain in clinical trials [29,30].

In the past decade, a number of studies have investigated the effect of BVA against chemotherapy-induced neuropathic pain, and different mechanisms of action have been proposed [31,32]; however, most of them focused on the spinal level. Therefore, its effect on the peripheral nervous system remains unclear. Moreover, to date, no studies have assessed its direct effect on acutely dissociated DRG neurons.

Thus, first, we aimed to assess the effect of oxaliplatin on voltage-dependent Na^+^ currents in acutely dissociated myelinated and unmyelinated DRG neurons in this study. Second, we confirmed that different doses of BVA administered at ST36 could significantly attenuate oxaliplatin-induced neuropathic pain in rats. Finally, we analyzed the possible mechanism of BVA in decreasing the pain transmission by conducting whole-cell current patch-clamp experiments in DRG neurons.

## 2. Results

### 2.1. Single Oxaliplatin Administration Induced Cold and Mechanical Allodynia in Rats

**Figure 1:**
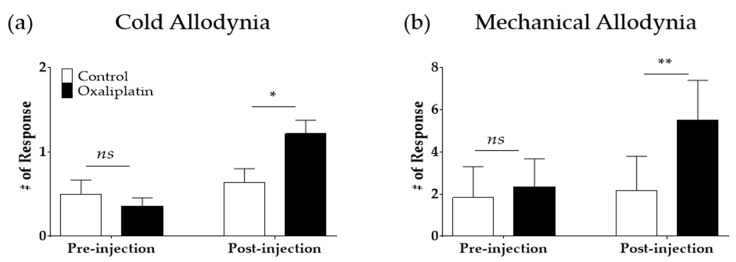
Single intraperitoneal injection of 6 mg/kg of oxaliplatin induced cold (**a**) and mechanical (**b**) allodynia in rats. The acetone and von Frey tests were used to assess cold and mechanical allodynia, respectively. Pre-injection: prior to the injection of oxaliplatin or 5% glucose (control). Post-injection: four days after the administration of oxaliplatin or 5% glucose. Control: *n* = 6, Oxaliplatin: *n* = 7. *ns*: Not significant, * *p* < 0.05, ** *p* < 0.01, vs. Control with two-way ANOVA followed by Sidak’s post-test for multiple comparisons.

A single intraperitoneal injection of oxaliplatin (6 mg/kg) elicited cold and mechanical allodynia (Figure 1). Cold and mechanical allodynia were measured by applying an acetone drop and von Frey filament (bending force of 0.4 g) on the mid-plantar hind paw, respectively. Before the injection of oxaliplatin or 5% glucose (control), rats in both groups showed no cold or mechanical hypersensitivity (pre-injection). However, four days after oxaliplatin injection, both cold (*p* < 0.05) and mechanical (*p* < 0.01) allodynia were significantly induced compared to the control group (post-injection).

### 2.2. Oxaliplatin Modulates Voltage-Dependent Na^+^ Currents in A- but Not C-fiber DRG Neurons 

**Figure 2:**
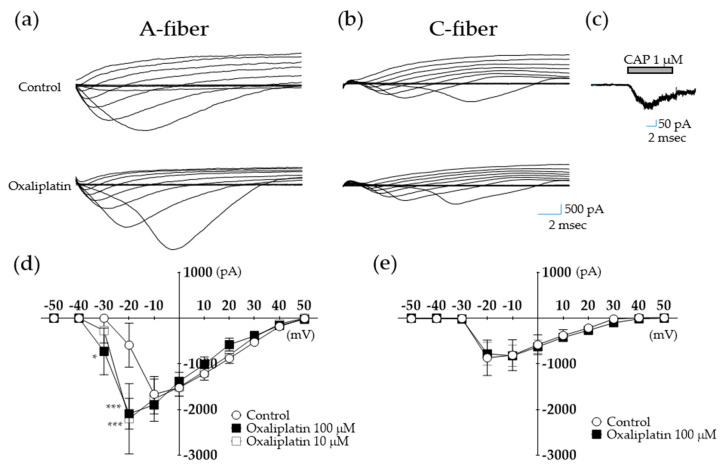
Effect of oxaliplatin on A- (**a**,**d**) and C- (**b**,**e**) fiber voltage-gated Na^+^ currents. Traces of inward Na^+^ currents recorded during depolarizing voltage steps from −60 to +50 mV at 10 mV increments with duration of 150 ms, before (control) and after the addition of either 10 or 100 µM of oxaliplatin to the external solution. Capsaicin (CAP, 1 μM) was used to identify A- and C-fiber neurons (**c**). Figure 2d: Control (external solution); *n* = 11, Oxaliplatin 100 μM; *n* = 11, Oxaliplatin 10 μM; *n* = 4. Figure 2e: Control (external solution); *n* = 5, Oxaliplatin 100 μM; *n* = 5. * *p* < 0.05, *** *p* < 0.001, vs. Control with two-way analysis of variance followed by Sidak’s post-test for multiple comparisons.

To observe the effect of oxaliplatin on the function of voltage-dependent Na^+^ currents, a depolarization step from − 60 to + 50 mV at 10 mV increments with a duration of 150 ms was injected in acutely dissociated A- and C-fiber DRG neurons (Figure 2a,d and Figure 2b,e, respectively). Representative images of changes in inward Na^+^ currents before (control) and after (oxaliplatin) treatment with oxaliplatin 100 μL in A- and C-fibers are shown (Figure 2a and Figure 2b, respectively). The peak current–voltage (I–V) curves are shown in Figure 2d,e. An artificial external recording solution was used as a control. Oxaliplatin treatment (100 μM) affected the inward Na^+^ currents in A-fibers but not in C-fiber neurons. Inward Na^+^ currents were observed at −30 mV in oxaliplatin-treated cells, whereas the 10 μM-treated cells did not show a significant change. When the step voltage was increased to −20 mV, both 10 and 100 μM showed significant differences compared to the control group (*** *p* < 0.001). These results show that oxaliplatin shifted the voltage-dependence of sodium conductance toward negative membrane potentials in A-fiber neurons. This effect of oxaliplatin could not be reversed even at prolonged washout times, as reported in another study [33]. In contrast to A-fiber sensory neurons, the function of the voltage-dependent sodium channel in C-fiber neurons remained unaffected after 100 μM of oxaliplatin at all voltages. To identify the type of neurons, the size of neurons and response to capsaicin application have been observed after each experiment. Capsaicin, the pungent component of chili pepper, is known to induce dose-dependent inward Na^+^ currents after its application [34], and 1 μM of capsaicin was selected as C-fiber neurons showed the highest response rate. Representative traces of C-fiber neuron responding to 1 μM of capsaicin treatment are shown in Figure 2c.

### 2.3. Oxaliplatin Lowers the Current Threshold for Action Potential Generation in A-Fiber DRG Neurons

**Figure 3:**
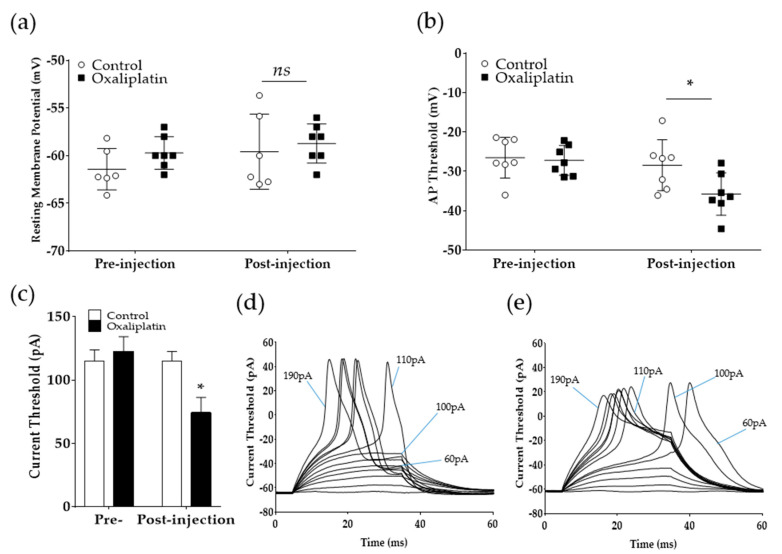
Effect of oxaliplatin on the resting membrane potential and action potential generation in A-fiber DRG neurons. In current patch-clamp analysis, the resting membrane potential was recorded. Action potentials were evoked using depolarizing step current injections of 10 pA from a set holding membrane potential of −60 mV. Resting membrane potential (**a**) and action potential threshold (**b**) in A-fiber DRG neurons before (pre-injection) and after (post-injection) the treatment of external solution or oxaliplatin (100 μM). Averaged current threshold for all cells tested (**c**). Representative traces of action potential generation after current injection before (**d**) and after oxaliplatin administration (**e**). Control (external solution): *n =* 6–7, oxaliplatin (100 μM): *n* = 7. *ns*: Not significant, * *p* < 0.05 vs. Control with two-way analysis of variance followed by Sidak’s post-test for multiple comparisons.

As we have observed, oxaliplatin could modulate the inward Na^+^ currents in the previous experiments. Consequently, the current patch-clamp recording method was used to assess the effect of oxaliplatin on the excitability of A-fiber neurons. Step currents of 10 pA increments for 30 ms were injected to induce action potentials in A-fiber DRG neurons (Figure 3). The results show that the resting membrane potential did not change following oxaliplatin treatment, whereas the threshold for action potential initiation (−28.45 ± 2.44 mV (control) vs. −35.78 ± 2.03 mV (oxaliplatin); *p* < 0.05) and the amount of current needed to generate an action potential (115 ± 7.63 pA (control) vs. 74.28 ± 12.12 pA (oxaliplatin); *p* < 0.05) significantly decreased. Representative action potential traces recorded from A-fiber neurons before and after oxaliplatin treatment are shown in Figure 3d,e. Before the administration of oxaliplatin, DRG neurons showed subthreshold responses to 60–100 pA, and an action potential was generated after 110 pA injection (current threshold for this neuron). However, after oxaliplatin application, 60–100 pA current injection evoked an action potential (Figure 3e).

### 2.4. Anti-Allodynic Effects of BVA against Oxaliplatin-Induced Cold and Mechanical Allodynia

**Figure 4:**
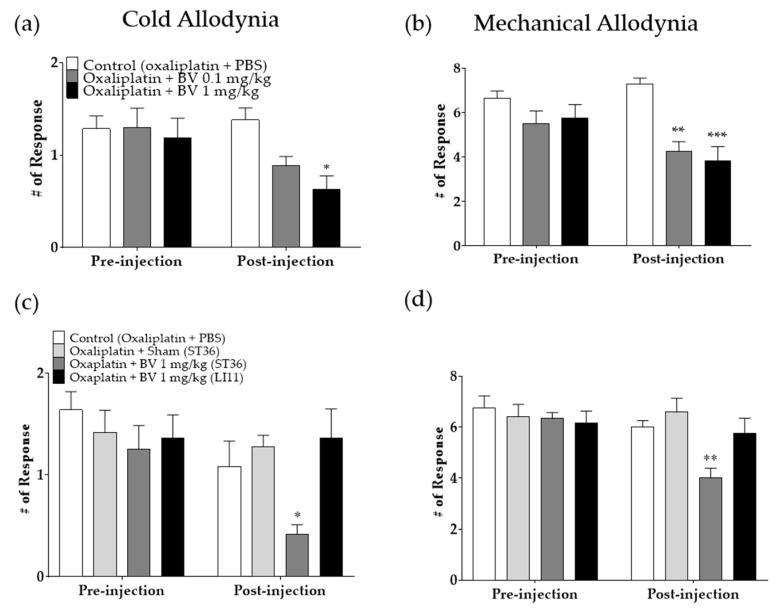
Single subcutaneous administration of bee venom acupuncture (BVA) at ST36 decreased cold (**a**,**c**) and mechanical (**b**,**d**) allodynia induced by oxaliplatin in rats. Cold and mechanical allodynia were assessed using acetone and von Frey tests, respectively. In all groups, behavioral assessments were conducted four days after the injection of 6 mg/kg of oxaliplatin (pre-injection), when significant allodynic signs were observed in rats and 30 min after the administration of phosphate-buffered saline (PBS) or 0.1 or 1 mg/kg of BVA or sham (brief syringe insertion) (post-injection). Control: *n* = 7, BV 0.1 mg/kg: *n* = 10, BV 1 mg/kg: *n* = 10 (**a**,**b**), Control: *n* = 6, Sham: *n* = 6, BV ST36: *n* = 6, BV LI11: *n* = 6 (**c**,**d**). * *p* < 0.05, ** *p* < 0.01, *** *p* < 0.001 vs. Control with two-way analysis of variance followed by Sidak’s post-test for multiple comparisons.

On day four, following the oxaliplatin injection, when the cold and mechanical allodynia were significantly induced, two different doses of BVA (0.1 and 1 mg/kg) or phosphate-buffered saline (PBS, control) were subcutaneously injected in the right leg of the rats (ST36, Figure 4). The site of injection was chosen as in our previous study, and a significant effect was shown compared to other sites [35]. Cold (Figure 4a,c) and mechanical (Figure 4b,d) allodynia induced by oxaliplatin injection (pre-injection) were significantly alleviated 30 min after the treatment with 1 mg/kg of BV compared to control (post-injection; *p* < 0.01 and *p* < 0.001 for cold and mechanical allodynia, respectively). However, 0.1 mg/kg of BV was effective against mechanical allodynia (*p* < 0.05) but not cold allodynia. Furthermore, to verify whether the anti-allodynic effect of BVA is acupoint specific, 1 mg/kg of BVA was administered at ST36 and LI11 (Figure 4c,d). In addition, the syringe used to treat with BVA was briefly (3 s) inserted in ST36 (oxaliplatin + sham (ST36)) to demonstrate that the effect of BVA was not induced by syringe application. The results show that only BVA at ST36 possess significant analgesic effect against cold and mechanical allodynia induced by oxaliplatin, whereas animals in other three group failed to show any sign of alleviation. BVA administered at ST36 in naïve rats did not induced any significant behavioral change compared to PBS-injected animals (Supplementary Materials Figure S1).

### 2.5. BVA Increases the Action Potential and Current Threshold in Acutely Dissociated DRG Neurons

**Figure 5:**
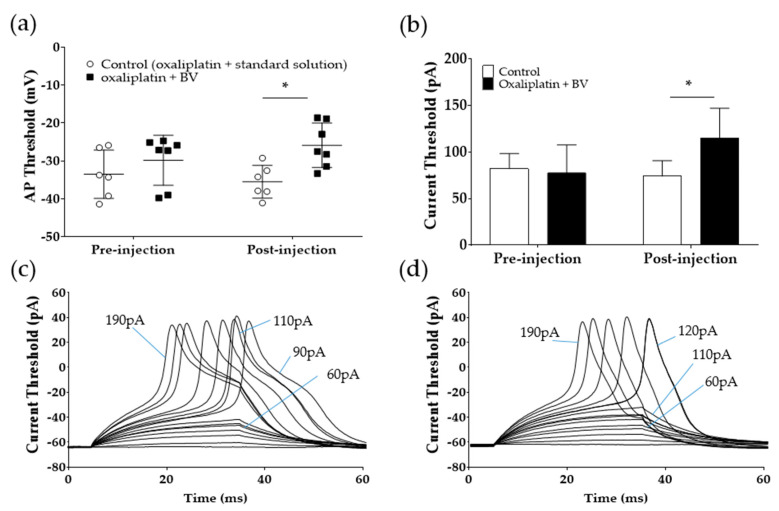
Effect of oxaliplatin on the current threshold for action potential generation in DRG neurons. Action potentials were evoked by using depolarizing step current injections of 10 pA from a set holding membrane potential of −60 mV. Action potential (**a**) and current threshold (**b**) in A-fiber DRG neurons before (pre-injection) and after (post-injection) the treatment of external solution or BV (0.1 μg/mL). Averaged current threshold for all cells tested (b). Representative traces of action potential generation after current injection before (**c**) and after 0.1 mg/kg BV administration (**d**). Control (external solution): *n* = 5, BV (0.1 μg/mL): *n* = 8. * *p* < 0.05 vs. Control with two-way analysis of variance followed by Sidak’s post-test for multiple comparisons.

As BVA significantly attenuated cold and mechanical allodynia in vivo, in our next experiments, we applied BV to acutely dissociated A-fiber DRG neurons to observe whether it can increase the lowered action potential threshold after oxaliplatin treatment. The current patch-clamp technique was used for this experiment. Application of 0.1 μg/mL of BV significantly increased the lowered action potential threshold of A-fiber DRG neurons after oxaliplatin injection (−35.52 ± 1.75 mV (control) vs. −25.86 ± 2.20 (oxaliplatin); *p* < 0.05) (Figure 5a). Furthermore, the currents needed to evoke action potential significantly increased after the BV treatment (74 ± 7.48 pA (control) vs. 115 ± 11.33 pA (oxaliplatin); *p* < 0.05) (Figure 5b). Representative traces from DRG neuronal cells show that after oxaliplatin treatment, 90 to 110 pA current injection evoked an action potential (Figure 5c), whereas after BV application, the current threshold needed to generate action potential increased to 120 pA (Figure 5d).

## 3. Discussion

In this study, first, by conducting both behavioral tests and voltage and current patch-clamp experiments, we showed that a single intraperitoneal oxaliplatin injection (6 mg/kg) can induce cold and mechanical allodynia in rats and modulate voltage-dependent Na^+^ currents in acutely dissociated A- but not C-fiber DRG neurons. The action potential threshold also decreased. Second, we demonstrated that subcutaneous administration of BVA at the right hind leg (0.1 and 1 mg/kg, ST36) could attenuate both cold and mechanical allodynia in a dose-dependent manner in rats. Finally, we demonstrated that 0.1 μg/mL BV could decrease the pain signal transmission to the spinal cord by increasing the lowered action potential threshold following oxaliplatin (100 μM) treatment in myelinated DRG neurons.

In the present study, cold and mechanical allodynia were induced by a single injection of oxaliplatin. It has been reported that intraperitoneal injection of 6 mg/kg of oxaliplatin can mimic the acute allodynia observed in humans [36]. Moreover, other studies demonstrated that oxaliplatin treatment can cause rapidly developing pain signs in more than 85% of the patients, with peaks in the first 24–48 h after the injection [3]. The cause of this acute toxicity is not clearly understood, but alteration in Na_v_ channels that leads to peripheral-nerve hyperexcitability is suggested to be the cause [37].

Recently, many studies have observed the effect of BVA on different types of diseases [38]. However, to date, this is the first study to evaluate its effect on acutely dissociated DRG neurons after chemotherapeutic agent administration. Previous studies have mostly focused on its action on the spinal cord [7,32,35]. Conversely, understanding the effect of BVA on the DRG is important as the major site affected by oxaliplatin appears to be the DRG [39]. Postmortem histopathological analysis in humans showed that platinum levels were highest in the DRG and lowest in the area protected by the BBB [40] implying the importance of DRG in oxaliplatin-induced neuropathic pain. 

DRG contain cell bodies of myelinated and unmyelinated sensory neurons (i.e., A- and C-fibers, respectively). To clarify which fiber type is affected by oxaliplatin, we assessed changes in voltage-dependent inward Na^+^ currents in both A- and C-fibers. Compared to the control, 100 μM of oxaliplatin application induced a negative shift in Na^+^ current activation (i.e., −30 vs. −20 mV (control)), whereas no difference was observed in C-fibers, showing that A- but not C-fiber DRG neurons are affected by oxaliplatin. These results corroborate two other studies conducted on human and animal nerves [33,41]. They showed that the function of A- but not C-fibers was affected by oxaliplatin, suggesting that A-fibers play a critical role in the transmission of oxaliplatin-induced pain. Although the specific subtype of A-fiber neurons was not elucidated in our study, it has been reported that Aδ-fibers, which are the thinnest myelinated fibers, may play an important role in oxaliplatin-induced neuropathic pain. Aδ-fibers are involved in the transduction of paresthesia (tickling or tingling sensations in the skin, whether distal or perioral), which is one of the most frequent symptoms of oxaliplatin-treated patients [42] as well as in cold pain [43].

In our subsequent experiments conducted using the whole-cell current patch-clamp technique, oxaliplatin significantly lowered the action potential threshold in A-fiber neurons, showing that an action potential could easily be generated after oxaliplatin administration. Accordingly, the current needed to generate the action potential also decreased. On the contrary, the resting membrane potential remained unchanged following oxaliplatin injection. These results show that membrane potential can be modulated at the depolarization level, and Na_v_ channels may be involved, as it was reported to modify the action potential threshold. Previously, Ruth et al. [41] reported that voltage-gated sodium channel 1.6 (Na_v_1.6) is involved in oxaliplatin-induced neuropathic pain. In their experiments, SCN8A^med/med^ mice, which lacked the function of Na_v_1.6, showed no sign of cold allodynia. Deuis et al. [44] also demonstrated that intraplantar injection of μ-conotoxin (GIIIA, 10 μM), which is able to inhibit Na_v_1.6, achieved near-complete reversal of oxaliplatin-induced cold allodynia. Na_v_1.6 is a tetrodotoxin-sensitive Na_v_ channel expressed by both myelinated and unmyelinated DRG neurons. In A-fibers, they are located at the node of Ranvier, whereas they are expressed in a continuous fashion along the axon of unmyelinated C-fibers [45].

In the present study, the role K_v_ channels was been investigated. Other studies have suggested a critical role of K_v_ channels in oxaliplatin-induced pain as 4-aminopyridine, which is a classic antagonist of K_v_ channels, showed similar effect to oxaliplatin in rodents [25]. Furthermore, Mannelli et al. [46] reported that single subcutaneous administration of hydrogen sulfide donors could reduce the hypersensitivity to cold noxious stimuli in a chronic animal model of oxaliplatin-induced neuropathic pain. They were shown to possess longer inhibitory effect compared to duloxetine and pregabalin, and the effect of hydrogen sulfide donors were shown to be mediated by K_v_7 channels, as its blocker nullified its analgesic effect. However, Benoit et al. [47] reported that the effect of oxaliplatin on Na_v_ channel appears to be greater than that on K_v_ channel. In addition, by measuring the compound action potential in rat sural, vagal, and peroneal nerves, Adelsberger et al. [33] reported that oxaliplatin did not block K^+^ conductance but altered the activation and inactivation behavior of Na^+^ channels. They further stated that the lengthening of action potential caused by oxaliplatin injection might be due to the slowdown of Na^+^ channel inactivation kinetics but not from the blockade of K^+^ channels. Thus, the prolonged repolarization phase seen in this study (Figure 3d) might also be due to the slowdown of Na_v_ channel inactivation kinetics, but future studies are needed to understand the precise role of Na_v_ and K_v_ channels in increase in the duration of action potential induced by oxaliplatin.

In this study, the dose-dependent relationship of BV was not measured as cells became easily unstable when BV at doses higher than 0.1 μg/mL (i.e., 0.3 and 1 μg/mL) was applied (data not shown). As mentioned previously, the effect of BV on acutely associated DRG neurons has never been observed before; however, the cytotoxic effect of BV has been analyzed in cultured cells. By using a 3-(4,5-dimethylthiazol-2-yl)-2,5-diphenyltetrazolium bromide cytotoxicity assay, rat brain cortical neurons showed cellular cytotoxicity with a 24 h treatment of 0.8 to 3 μg/mL BV, whereas 0.2 or 0.4 μg/mL BV did not show any cellular toxicity [48]. In the human lung carcinoma cell line NCI-H1299, 1 μg/mL of BV showed cytotoxic effects after 12 h of treatment. However, there may be a difference in the properties between acutely dissociated neurons and cultured cells. Conducting experiments on acutely dissociated animal DRG may have more clinical relevance than those on cultured cells, as differences in electrophysiological properties were observed, such as changes in the action potential size and reduced voltage-gated Na^+^ currents along with its corresponding mRNA expression [49].

## 4. Conclusions 

In conclusion, our study demonstrated that BVA injection could attenuate oxaliplatin-induced neuropathic pain by increasing the lowered action potential threshold. Future studies are needed to elucidate the action of BVA on the peripheral nervous system. The action of BVA on the function of various ion channels present in the DRG, such as potassium and calcium ion channels, should be investigated.

## 5. Materials and Methods 

### 5.1. Animals

Sprague-Dawley (SD) rats (post-natal 10-18 days (for electrophysiological studies) and 6 weeks old (for behavioral tests)) were obtained from the supplier (DBL, Chungbuk, Korea) and housed in an specific pathogen free (SPF) animal center. Animals were randomly caged with a temperature of 23 ± 2 °C, humidity of 65% ± 5%, with food and water ad libitum, and a fixed 12 h light/dark cycle. All experimental protocols were approved by the Kyung Hee University Animal Care and Use Committee (KHUASP(SE)-20-147) on 12 May 2020.

### 5.2. Administration of Drugs

Oxaliplatin (Sigma Aldrich, St. Louis, MO, USA) was dissolved in 5% glucose solution at a concentration of 2 mg/mL. Oxaliplatin was administered intraperitoneally at a dose of 6 mg/kg. Capsaicin (Sigma Aldrich, St. Louis, MO, USA) was dissolved in ethanol at a concentration of 1 μM. BV was manufactured by Jayeonsaeng TJ (Gyeonggi-Do, Korea), controlled by regular high-performance liquid chromatography (HPLC) analysis (SNU National Instrumentation Center for Environmental Management, Seoul, Korea) [35]. BV was dissolved in PBS at a concentration of 1 mg/mL. BVA was subcutaneously injected into acupoint ST36 (right hind leg) or LI11 (located at the depression medial to the extensor carpi radialis, at the lateral end of the cubital crease) at doses of 0.1 or 1 mg/kg. A BD ultra-fine II short needle insulin syringe −31G (Becton, Dickinson, and company, NJ, USA) was used to inject BVA subcutaneously. In a previous study, 0.1 or 1 mg/kg of BVA was reported to be an effective concentration causing no side effects. Acupoint ST36 was chosen as it was shown to be effective compared to other sites in our previous studies [35].

### 5.3. Behavioral Tests

Behavioral tests were conducted as described in our previous studies [7,9]. Briefly, for acclimation, mice were caged in an inverted clear plastic cage (12 × 8 × 6 cm) on a metal mesh floor for 30 min prior to the measurements. Cold allodynia was assessed by gently applying a drop of acetone (10 μL, Daejung Chemical Ltd., Gyeonggi-do, Korea) to the mid-plantar skin (avoiding the base of the tori) of both hind paws (three times). The frequencies of brisk withdrawal, licking, and shaking of the hind paw were counted for 30 s as a sign of pain. Thus, “# of Response” in Figure 1a and Figure 4a,c indicates the average number of responses to 10 μL of acetone drop counted for 30 s.

Mechanical allodynia was assessed using the von Frey filament (Linton Instrumentation, Norfolk, U.K.) test. Mice were placed on the same wire mesh floor covered with a clear plastic box as mentioned above, and a von Frey filament with a bending force of 0.4 g (3.61 log scale) was applied to the point of bending ten times to the mid plantar skin of each hind paw. Stimulation was applied at intervals of 3 s, and lifting the paw was counted as a sign of pain. The “# of Response” in Figure 1b and Figure 4b,d represent the average number of paw-lifting instances to multiple von Frey filament stimulation (10 times).

### 5.4. Acute Dissociation of DRG Neurons

DRG neurons were dissociated using procedures previously described elsewhere [50]. Briefly, in deeply anesthetized (urethane, Sigma Aldrich, St. Louis, MO, USA) SD rats, the lumbar 4–6 DRG were separated from the surrounding tissue and immediately placed into a Petri dish containing 4 °C of artificial cerebrospinal fluid (ACSF), which was composed of the following (in mM): 125 NaCl, 3 KCl, 1.2 KH_2_PO_4_, 1.2 MgSO_4_, 25 NaHCO_3_, 10 glucose, and 2 CaCl_2_, and bubbled with 95% O_2_–5% CO_2_. After removing excess connective tissues, the DRG was incubated for 30–45 min at 31 °C in an ACSF solution containing protease (type 2S trypsin, 5 to 6 mg/mL at 1310 U/mg; Sigma Aldrich). After protease treatment, the ganglion was placed in enzyme-free ACSF for 1–2 h at room temperature. The ganglia were mechanically dispersed using fire-polished Pasteur pipettes in a glass-bottom perfusion chamber filled with an artificial external recording solution containing (in mM): 150 NaCl, 5 KCl, 1 MgCl_2_, 2 CaCl_2_ 10 N-2-hydroxyethylpiperazine-N’-2-ethanesulfonic acid (HEPES); 10 glucose (pH was adjusted to 7.4 with Tris base).

### 5.5. Whole Cell Voltage and Current Patch-Clamp

For voltage and current patch-clamp recordings, isolated neurons were visualized using a phase contrast microscope (Eclipse Ti; Nikon, Tokyo, Japan). Recordings were performed using a Multiclamp 700B (Molecular Devices, Sunnyvale, CA, USA) and pClamp 9 software (Molecular Devices). Recording electrodes were back-filled with an intracellular solution containing the following (in mM): 10 NaCl, 130 K-gluconate, 11 EGTA, 1 CaCl_2_, 2 MgCl_2_, and 10 HEPES. Immediately before recording, 2 mM Na-ATP and Na-GTP were added to the pipette solution. The pH was adjusted to 7.3 using 1N KOH. During the experiments, the pipette solution was kept from light and chilled (8–12 °C). The osmolarities of the pipette solutions were adjusted to 260 and 267 mOsm. All experiments were conducted at room temperature (21–23 °C).

### 5.6. Statistical Analysis

All data are presented as mean ± standard error of the mean (SEM). Electrophysiological data were analyzed offline using the pClamp 9 software. Currents were sampled at 10 kHz with 3 kHz filtering. Statistical analysis and graphic works were performed using Prism 7.0 (GraphPad software, La Jolla, CA, USA). Two-way ANOVA followed by Sidak’s post-test for multiple comparisons was used for statistical analysis. In all cases, *p* < 0.05 was considered significant.

## Supplementary Materials

The following are available online at https://www.mdpi.com/2072-6651/12/12/737/s1, Figure S1: BVA at ST36 does not affect responses to cold and mechanical stimulation in naïve rats.
