# Supplementary material for: Bee Venom Acupuncture Attenuates Oxaliplatin-Induced Neuropathic Pain by Modulating Action Potential Threshold in A-Fiber Dorsal Root Ganglia Neurons
Source: Toxins (Basel). 2020 Nov 24;12(12):737. doi: 10.3390/toxins12120737 (PMC7760131; doi:10.3390/toxins12120737)
Supplement: Supplementary file 1 [file toxins-12-00737-s001.pdf]

Supplementary Materials: Bee Venom Acupuncture Attenuates Oxaliplatin-Induced Neuropathic Pain by Modulating Action Potential Threshold in A-fiber Dorsal Root Ganglia Neurons

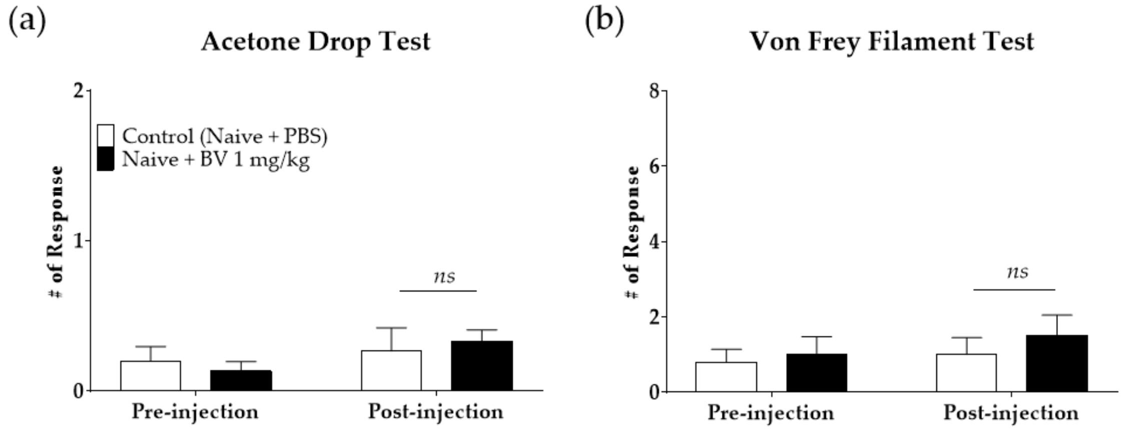

Figure S1. BVA at ST36 does not affect responses to cold and mechanical stimulation in naïve rats.Bee venom acupuncture (BVA) administration at ST36 does not modify the behavioral responses to acetone drop (**a**) and von Frey filament (**b**) in naïve rats. Acetone drop (10 µl) or von Frey filament (0.4 g bending force) was applied to the mid-plantar skin to mimic the behavioral tests conducted to assess the cold and mechanical allodynia after oxaliplatin injection, respectively. Control group only received PBS at ST36 (Naïve + PBS), whereas experimental group received 1 mg/kg of BVA at ST36 (Naïve + BV 1 mg/kg). In all groups, behavioral assessments were conducted four days after acclimation. Control: $n = 5$, Naïve + BV 1 mg/kg: $n = 5$. Non significant (*ns*) *vs.* control with two-way ANOVA followed by Sidak's post-test for multiple comparisons.
